# Generation of Markerless Deletions in the Nosocomial Pathogen *Clostridium difficile* by Induction of DNA Double-Strand Breaks

**DOI:** 10.1128/AEM.02055-18

**Published:** 2019-01-23

**Authors:** Elena-Stella Theophilou, Prerna Vohra, Maurice P. Gallagher, Ian R. Poxton, Garry W. Blakely

**Affiliations:** a Institute of Quantitative Biology, Biochemistry and Biotechnology, University of Edinburgh, Edinburgh, United Kingdom; b Microbial Pathogenicity Research Laboratory Medical Microbiology, University of Edinburgh, Edinburgh, United Kingdom; c The Roslin Institute and Royal (Dick) School of Veterinary Studies, University of Edinburgh, Edinburgh, United Kingdom; University of Tartu

**Keywords:** AddAB, *Clostridium difficile*, deletions, nosocomial, SOS system, allelic exchange, double-strand-break repair, *fliC*, mutagenesis

## Abstract

*Clostridium difficile* is an important nosocomial pathogen associated with potentially fatal disease induced by the use of antibiotics. Genetic characterization of such clinically important bacteria is often hampered by lack of availability of suitable tools. Here, we describe the use of I-SceI to induce DNA double-strand breaks, which increase the frequency of allelic exchange and enable the generation of markerless deletions in *C. difficile*. The usefulness of the system is illustrated by the deletion of genes encoding putative AddAB homologues. The Δ*addAB* mutants are sensitive to ultraviolet light and the antibiotic metronidazole, indicating a role in homologous recombination and the repair of DNA breaks. Despite the impairment in recombination, the mutants are still proficient for induction of the SOS response. In addition, deletion of the *fliC* gene, and subsequent complementation, reveals the importance of potential regulatory elements required for expression of a downstream gene encoding the flagellin glycosyltransferase.

**IMPORTANCE** Most sequenced bacterial genomes contain genes encoding proteins of unknown or hypothetical function. To identify a phenotype for mutations in such genes, deletion is the preferred method for mutagenesis because it reduces the likelihood of polar effects, although it does not eliminate the possibility. Allelic exchange to produce deletions is dependent on the length of homologous regions used to generate merodiploids. Shorter regions of homology resolve at lower frequencies. The work presented here demonstrates the utility of inducing DNA double-strand breaks to increase the frequency of merodiploid resolution in *Clostridium difficile*. Using this approach, we reveal the roles of two genes, encoding homologues of AddAB, in survival following DNA damage. The method is readily applicable to the production of deletions in *C. difficile* and expands the toolbox available for genetic analysis of this important anaerobic pathogen.

## Abstract (precis)

Most sequenced bacterial genomes contain genes encoding proteins of unknown or hypothetical function. To identify a phenotype for mutations in such genes, deletion is the preferred method for mutagenesis because it reduces the likelihood of polar effects, although it does not eliminate the possibility. Allelic exchange to produce deletions is dependent on the length of homologous regions used to generate merodiploids. Shorter regions of homology resolve at lower frequencies. The work presented here demonstrates the utility of inducing DNA double-strand breaks to increase the frequency of merodiploid resolution in *Clostridium difficile*. Using this approach, we reveal the roles of two genes, encoding homologues of AddAB, in survival following DNA damage. The method is readily applicable to the production of deletions in *C. difficile* and expands the toolbox available for genetic analysis of this important anaerobic pathogen.

## INTRODUCTION

*Clostridium difficile*, also known as *Clostridioides difficile* (1), is a Gram-positive obligately anaerobic spore-forming bacillus originally isolated in 1935 from the fecal microbiota of healthy neonates (2). *C. difficile* was first associated with human infections in 1962 (3), but its role as the pathogen responsible for antibiotic-associated pseudomembranous colitis was not confirmed until the late 1970s (4–6). *C. difficile* is currently one of the most commonly reported pathogens in nosocomial infections in the United States and the European Union (7–11). The bacterium is acquired through ingestion of vegetative cells or spores, which are ubiquitous in the environment, and although not part of the normal gut microbiota of humans, 1% to 3% of adults are carriers (12). Exposure to antibiotics is a major risk factor for *C. difficile* infection (CDI) (13, 14). Disruption of the normal gut microbiota leads to a loss of colonization resistance which, together with spore germination due to exposure to bile salts, results in proliferation of *C. difficile* (15–17). The organism adheres to the mucus layer covering the epithelial surface of the gastrointestinal (GI) tract via multiple adhesins, and then it penetrates the mucus and adheres to enterocytes, marking the beginning of the first phase of the pathogenic process (18, 19). The second important phase of pathogenesis is toxin production (18, 19). Toxigenic *C. difficile* strains produce two major toxins, toxin A (TcdA) and toxin B (TcdB), which are encoded on a 19.6-kb chromosomal region termed the pathogenicity locus (PaLoc) and are recognized as primary virulence factors (20, 21).

Although treatment of CDI depends on the clinical presentation of disease (13, 22), the first step is usually the discontinuation of the inciting antibiotic. Metronidazole is the first choice for mild to moderate CDI (23), while vancomycin is preferred as the first-line drug for moderate to severe CDI (23, 24). Due to an increased rate of failure of metronidazole and vancomycin treatments and recurrence of CDI (25), alternatives, such as fidaxomicin, a narrow-spectrum antibiotic (26, 27), rifaximin, a broad-spectrum nonabsorbable antibiotic (28), and nitazoxanide, a broad-spectrum antiparasitic (29), have been tested against *C. difficile*.

Metronidazole targets the DNA of bacterial cells. The antibiotic enters cells by passive diffusion, and the prodrug is activated in the cytoplasm. The molecule is converted to a nitroso free radical, which interacts with DNA to cause single- or double-stranded chromosomal breaks, resulting in DNA degradation and cell death (30, 31). In *Bacteroides fragilis*, resistance to metronidazole can result from the presence of *nim* genes, which inactivate the nitroso radicals causing DNA damage (32). Alternatively, resistance can be mediated by deficiency of the ferrous iron transporter FeoAB, which potentially interferes with metronidazole activation (33) or increased production of proteins involved in homologous recombination, such as RecA (34) and RecQ (35). Increased expression of other genes involved in DNA repair, such as *recF*, *recN*, and *uvrA*, has been observed during the growth of *B. fragilis* under subinhibitory concentrations of metronidazole, indicating the importance of recombination as a response to the drug (36). Similar mechanisms of resistance have also been described for *C. difficile*, including an increase in RecA production by metronidazole-resistant strains (37, 38).

The *C. difficile* genome has a large number of integrated and extrachromosomal mobile genetic elements, including conjugative and nonconjugative transposons and bacteriophages (39, 40), which illustrates the importance of horizontal gene transfer during the evolution of this bacterium (41, 42). Conjugative transposon-mediated transfer of the PaLoc from toxigenic to nontoxigenic *C. difficile* strains has also been described (43). It has been hypothesized that the chromosomal transfer and recombination events require the action of the relaxosome on the *oriT* of the conjugative transposon without prior excision of the element, a mechanism similar to the well-characterized Hfr conjugation mediated by an integrated F plasmid in *Escherichia coli*, albeit occurring at a much lower frequency (43). Integration of transferred chromosomal fragments requires homologous recombination with processing of double-stranded DNA ends to enable loading of RecA. However, little is currently known about homologous recombination in *C. difficile*.

**FIG 1:**
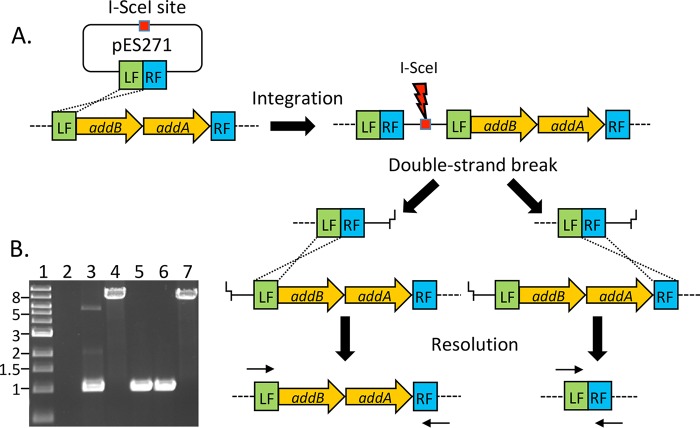
Schematic illustrating the generation of markerless deletions in *C. difficile* by induction of double-strand breaks. (A) DNA flanking the *addAB* genes (LF, left flank; RF, right flank) was amplified and ligated into a plasmid containing an I-SceI recognition sequence (pES271). Homologous recombination between the LF sequences on the plasmid and chromosome led to integration of the vector. The introduction of an I-SceI-expressing plasmid generates a double-strand break in the chromosome which must be repaired for the cell to survive. Recombination between LF sequences regenerates the original chromosome configuration of *addAB*, while recombination between RF sequences generates a deletion of the intervening DNA. Small arrows above LF and below RF represent the positions of primers used to differentiate between wild-type and deletion genotypes. (B) Example of an agarose gel showing PCR products for genotyping-resolved integrants. Primers used in the reaction correspond to the small arrows at LF and RF shown in panel A. The wild-type amplicon is 8.2 kb, while the deletion amplicon is ∼1 kb. Lane 1, 1-kb size ladder; lane 2, negative PCR control; lane 3, deletion amplicon from pES271; lane 4, wild-type *addAB* amplicon from 630Δ*erm*; lanes 5 and 6, products from two independent Δ*addAB* mutant strains; lane 7, product from resolved wild-type strain.

Reverse genetics requires methodologies for generating defined mutations. The lack of selectable markers in multidrug-resistant strains of bacteria poses a particular problem for tool development, while insertional inactivation of genes using antibiotic resistance makes the production of multiple mutations problematic. Markerless deletions are therefore preferred because they are less likely to produce polar effects, and the resistance determinant, used during construction, can be recycled for multiple rounds of mutagenesis. Deletions can be generated by allelic replacement, where a construct lacking the gene of interest is first integrated into the genome of the target strain (Fig. 1). Integrants can be selected based on antibiotic resistance; however, resolution of the merodiploid can be a rare event which makes the process potentially laborious. To increase the frequency of resolution events, a method originally described for *E. coli*, involving the yeast homing endonuclease I-SceI, can be used (44, 45). This approach utilizes site-specific cleavage by the endonuclease of an 18-bp cognate recognition site present in the vector carrying the construct of interest, resulting in loss of the vector at high frequencies and selecting for bacteria in which homologous recombination has successfully repaired the double-stranded break (Fig. 1). Induction of double-strand breaks has been used successfully in pathogens which were once considered genetically intractable, such as *Bacteroides fragilis* (46). In this study, the application of I-SceI-mediated DNA cleavage to create markerless mutants in *C. difficile* is described. Further, markerless mutants of *addAB* homologues and *fliC* in *C. difficile* are described to gain insight into homologous recombination in this bacterium and to demonstrate the utility of this method, respectively.

## RESULTS

### Enhancing allelic exchange using double-strand breaks.

To facilitate the generation of markerless mutants, an erythromycin-sensitive strain, *C. difficile* 630Δ*erm*, was used for mutagenesis. Sequence analysis of the parental strain (GenBank accession number AM180355) identified two adjacent genes annotated as encoding ATP-dependent helicase/DNase subunits, suggesting that they were involved in double-strand-break repair. The proteins encoded by genes CD630DERM_RS05940 and CD630DERM_RS05945 share 34% identity (399/1,182 residues) and 38% identity (484/1,279 residues) with AddB and AddA of *Bacillus subtilis*, respectively (here referred to as *addAB*). In most Gram-positive and many Gram-negative bacteria, the AddAB heterodimer is involved in degradation of double-stranded DNA as part of the presynaptic processing step of homologous recombination (47, 48).

Approximately 500-bp sequences flanking both sides of *addAB* were amplified and fused by PCR. The fusion product was inserted into an unstable conjugative vector, pES185, which is based on pJIR1456 (49) but modified to contain an I-SceI recognition sequence. The resulting plasmid containing the deletion construct, pES271, was conjugated into *C. difficile* 630Δ*erm* using the RP4 functions provided by *E. coli* S17-1λ*pir*. Transconjugants were selected on brain heart infusion (BHI) plates containing thiamphenicol, followed by analysis using PCR to confirm presence of the deletion construct present in the plasmid. Potential integrants resulting from a single crossover between the plasmid and chromosome were selected on thiamphenicol after multiple passages in broth without selection. Thiamphenicol-resistant integrants were screened by PCR using primers that annealed to plasmid and chromosomal DNA to generate a unique amplicon. Integration can occur either between sequences on the left flank (LF) or the right flank (RF) of the deletion construct. Note that Fig. 1A illustrates integration via a single crossover at the left flank.

To generate double-strand breaks, a plasmid containing the gene encoding I-SceI under the control of the constitutive *fdx* promoter from *Clostridium sporogenes* (50) was constructed by ligation into pMTL82254. This I-SceI-expressing plasmid, pES288, was introduced into merodiploids by conjugation, and erythromycin-resistant (Erm^r^) colonies were screened for sensitivity to thiamphenicol. The resolution frequency from three independent matings varied from 7% to 33%. In contrast, screening of 80 transconjugants with a vector that did not contain the gene encoding I-SceI failed to yield any thiamphenicol-sensitive strains following the same procedure. Thus, expression of I-SceI enhanced the second recombination event, which was required for resolution of the merodiploids.

### Phenotypic analysis of *C. difficile* Δ*addAB* mutants.

Recombination to resolve integrants should theoretically produce an equal ratio of wild-type to deletion genotypes, provided there are no confounding factors, such as the presence of chi sites or growth defects of the mutants which might affect the outcome. Screening by PCR of thiamphenicol-sensitive strains allowed the identification of mutants derived from the *addAB* merodiploid (Fig. 1B). However, the frequency of Δ*addAB* mutants (4% to 7%) present within all the resolved strains indicated some form of bias against the deletion genotype during or after the resolution event. Such a bias could reflect the frequency of chi sites in the sequences flanking the double-strand break; however, the sequence of potential chi sites in *C. difficile* has not been determined. Sequencing of PCR products spanning the *addAB* region in the deletion strains showed that 7.25 kb of DNA had been deleted precisely at the point defined by the sequence of the primers used to generate the construct in pES271 (data not shown).

Previous studies in other species have demonstrated growth defects in mutants defective in presynaptic processing of double-strand breaks. In *E. coli*, *recBC* mutants have reduced viability and increased doubling times (51). This is also true for *addAB* mutants of *Streptococcus pneumoniae* and *Bacteroides fragilis* (52, 53). The growth of two independently derived Δ*addAB* mutants was studied in anaerobic investigation medium (AIM) broth, and both strains had an average doubling time of ∼45 min, similar to that of the parental strain 630Δ*erm* (∼40 min). This suggests that in *C. difficile*, the deletion of *addAB* has a minor effect on viability.

**FIG 2:**
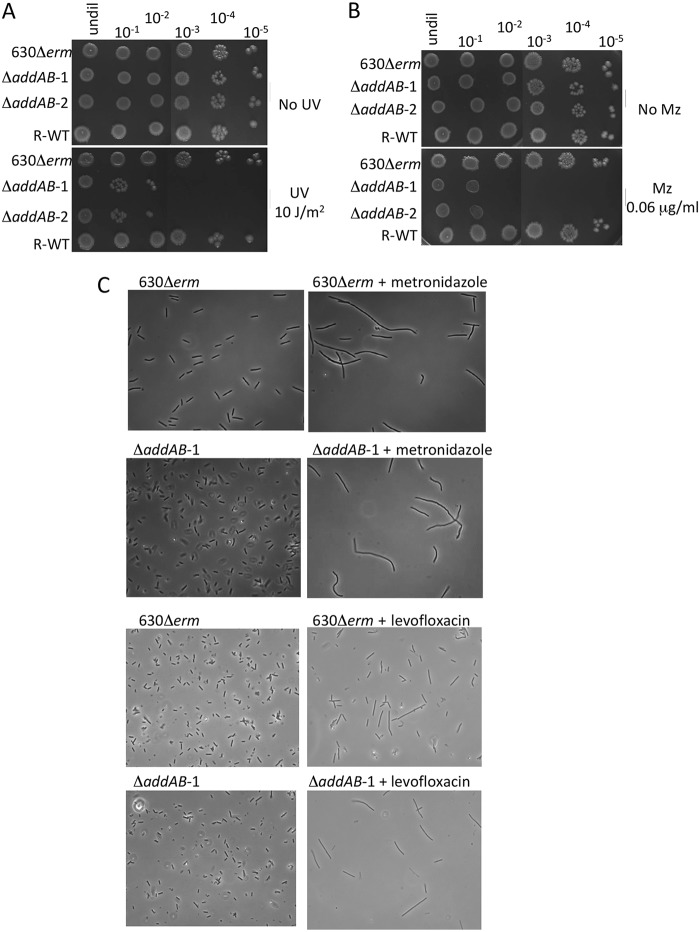
Phenotypic analysis of Δ*addAB* mutant strains. (A) Exponentially growing cultures of parental (630Δ*erm*), Δ*addAB* mutant (Δ*addAB*-1 and Δ*addAB*-2) and resolved wild-type (R-WT) strains were diluted, spotted onto AIM agar, and irradiated at 10J/m^2^ with UV light (bottom images). The no-UV control is shown in the top images. Dilution values are shown at the top of the panel. Note the similar viability of the parental and Δ*addAB* mutant strains in the absence of DNA damage. Undil, undiluted. (B) Exponentially growing cultures of all strains were diluted (shown at the top of the panel) and spotted onto AIM agar with and without 0.06 μg/ml metronidazole (Mz). (C) Micrographs showing random fields of cells (×400 magnification), illustrating the effect of 0.125 μg/ml metronidazole or 1 μg/ml levofloxacin on cell morphology for parental and Δ*addAB* mutant strains. The micrographs on the left show cells grown in the absence of antibiotics, while the images on the right show cell filamentation for both strains when metronidazole or levofloxacin was present.

The role of AddAB in DNA repair was tested by exposing cells to the DNA-damaging agents ultraviolet (UV) light and metronidazole. Exposure of the Δ*addAB* mutant strains to 10 J/m^2^ of UV resulted in a 1,000-fold decrease in viability, while exposure of the parental strain to the same dose of UV had little effect (Fig. 2A). A merodiploid that had resolved to produce the wild-type genotype (R-WT) behaved in a manner similar to the parental strain. Growth of the parental strain 630Δ*erm* on subinhibitory concentrations (0.06 μg/ml) of metronidazole was unaffected compared to that with absence of the antibiotic, but the growth of the two Δ*addAB* mutant strains was reduced by 1,000-fold in the presence of metronidazole (Fig. 2B).

The presence of single-stranded DNA resulting from inhibition of replication or following DNA damage leads to induction of the SOS response in many bacteria, including *C. difficile* (54). By microscopy, cultures of 630Δ*erm* grown in the presence of 0.125 μg/ml metronidazole for 4 h showed the presence of filamentous cells, consistent with division inhibition associated with the SOS response (Fig. 2C). Growth of the Δ*addAB* mutant strains in the same concentration of metronidazole also produced similar filamentous cells, suggesting that AddAB is not required for induction of the SOS response. To confirm that inhibition of division was due to the SOS response, we treated cells with levofloxacin, which has previously been shown to induce filamentation associated with a LexA-regulated pathway (54). Treatment of the 630Δ*erm* strain with a subinhibitory concentration of levofloxacin (1 μg/ml) for 6 h led to an increase in length of a subpopulation of cells (Fig. 2C). The average length of 630Δ*erm* cells in an untreated culture was 5.1 ± 0.99 μm, while the addition of levofloxacin increased the average cell length to 11.3 ± 8.9 μm. The addition of levofloxacin to the Δ*addAB* mutant strain had a similar effect (Fig. 2C). The average cell length of the Δ*addAB* mutant strain was 5.3 ± 1.6 μm, while treated cells had an average length of 18.8 ± 9.4 μm. Together, these data suggest that the AddAB homologues present in *C. difficile* are dispensable for induction of the SOS response.

### Deletion and complementation of *fliC*.

Despite the success of the deletion strategy, screening for integrants was a time-consuming process. To improve the efficiency of the system, another plasmid, pMTL83151, was used, which was reported to show segregational instability and therefore had the identifiable phenotype of two different colony sizes. In the presence of thiamphenicol, smaller colonies appear to result from a loss of plasmid in the population and so have fewer resistant cells, while larger colonies have a chromosomally integrated plasmid and are thus resistant to the antibiotic (55, 56). This plasmid was modified by introducing an I-SceI recognition sequence to produce vector pES242. To validate this approach, a deletion of the *fliC* gene (CD630DERM_RS01750), which encodes the major structural protein of the flagellum, was generated. This target was chosen because of the previously described loss of motility in a *C. difficile* strain in which the *fliC* gene had been insertionally inactivated (57).

**FIG 3:**
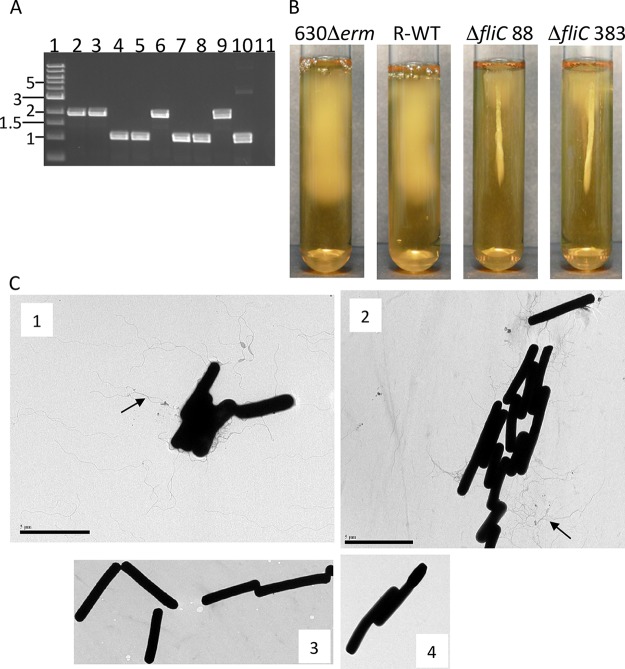
Identification and phenotypic analysis of Δ*fliC* mutants. (A) Example of an agarose gel showing PCR screening of resolved merodiploids. PCR primers used will amplify a 1.9-kb sequence which includes the *fliC* gene. Lane 1, 1-kb size ladder; lanes 2, 3, and 6, resolution to the wild type; lanes 4, 5, 7, and 8, deletions of *fliC*; lanes 9 and 10, controls amplifying the *fliC* region in 630Δ*erm* and the deletion construct; lane 11, PCR negative control. (B) Motility stab assay using 0.175% soft agar. The parental strain 630Δ*erm* and resolved wild-type (R-WT) strains showed penetration of the medium from the initial inoculum. Two independent *fliC* deletion mutants (88 and 383) grew at the site of inoculation but failed to spread into the agar. (C) Transmission electron micrographs of the parental strain (1) and a resolved wild-type strain (2) show the presence of thread-like flagella (examples indicated by arrows) associated with the cells. There was no evidence of flagella on the cell surfaces of either of the *fliC* mutants (3 and 4).

Approximately 500-bp sequences flanking both sides of *fliC* were amplified and fused by PCR, followed by cloning into pES242 to produce the allelic replacement vector pES2921. This plasmid was transferred into 630Δ*erm* by conjugation. When streaked onto thiamphenicol BHI plates, two colony sizes were evident, suggesting that integrants were present. Putative integrants were cultured in the absence of thiamphenicol and restreaked onto agar with thiamphenicol until no small colonies were evident. Integration of pES2921 was confirmed by PCR (data not shown). The I-SceI-expressing plasmid pES288 was introduced by conjugation, and Erm^r^ colonies were screened for loss of thiamphenicol resistance. The resolution frequencies of integrants derived from two independent matings were 2% (3/144) and 3.6% (4/111). The introduction of pMTL82254 into one of the integrants did not produce any thiamphenicol-sensitive colonies, again indicating the action of I-SceI on the merodiploids to enable resolution. Screening of resolved strains by PCR showed that approximately half the colonies were wild type, and half contained deletions of *fliC* (Fig. 3A). Motility assays in soft agar demonstrated that two independently derived Δ*fliC* mutant strains (strains 88 and 383) were incapable of penetrating the medium beyond the site of inoculation (Fig. 3B), a phenotype indicative of the deletion genotype. Transmission electron microscopy (TEM) of cells grown on AIM showed the production of peritrichous flagella (observed as numerous thread-like extensions from the cell surface) in the parental strain and an absence of flagella in the Δ*fliC* mutant strains (Fig. 3C), again confirming successful deletion of the target gene. In addition, we note that resolution of the integrants also produced cells that were motile and contained a wild-type copy of the *fliC* gene (Fig. 3B).

**FIG 4:**
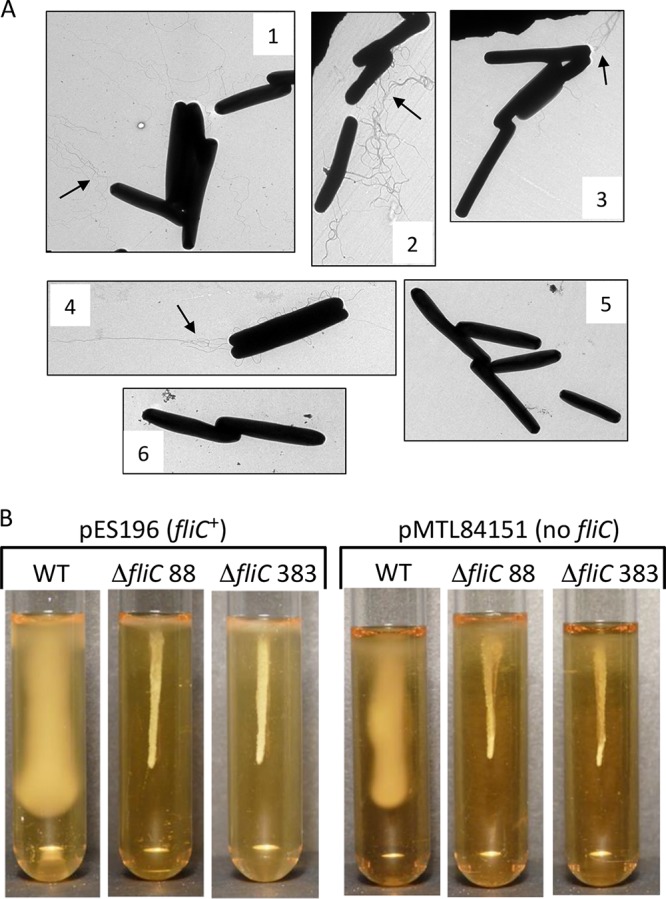
Complementation of Δ*fliC* mutants. (A) Transmission electron micrographs of strains containing either pES196 (*fliC*^+^) or the vector alone (pMTL84151). 1, 630Δ*erm*/pES196; 2, Δ*fliC* 383/pES196; 3, Δ*fliC* 88/pES196; 4, 630Δ*erm*/pMTL84151; 5, Δ*fliC* 383/pMTL84151; 6, Δ*fliC* 88/pMTL84151. Flagella are indicated by arrows. (B) Motility stab assay for strains containing either the *fliC* complementing plasmid (pES196) or the vector alone (pMTL84151). The strain genotype is indicated above each tube, with the presence of each plasmid shown at the top of each set of tubes. Only wild-type strains (630Δ*erm*) containing a chromosomally carried *fliC* gene showed penetration of the medium.

One potential advantage of markerless deletions, compared to insertion mutations, is the reduced possibility of polar effects on the expression of downstream genes. Since the *fliC* gene is within a gene cluster required for flagellar assembly and function, we tested complementation of the deletion using a plasmid-borne copy of the gene. The native *fliC* promoter and gene, as previously identified (57), were amplified and ligated into pMTL84151 to produce pES196, followed by conjugation of the plasmid into the Δ*fliC* mutant strains and the parental 630Δ*erm* strain. Electron microscopy showed visible production of flagella by the Δ*fliC* mutant strains and the parental strain when they contained pES196 (Fig. 4A). When the control plasmid pMTL84151 was present, flagella were only produced by the parental strain and not in the Δ*fliC* mutant strains. These data demonstrate successful complementation of the Δ*fliC* mutation by the presence of assembled flagella on the cell surface. Despite the observations made by TEM, motility assays in soft agar showed that the complemented Δ*fliC* mutant strains were not capable of penetrating the medium (Fig. 4B), and the inoculum had the same appearance as the stab tubes from the deletion strains (Fig. 3B). Microscopy of cells in a wet mount showed that Δ*fliC* cells containing pES196 had a tumbling behavior rather than the linear swimming motion of the parental strain (data not shown). Together, these data indicate that the flagella observed on the complemented Δ*fliC* mutant strains were functionally defective, possibly due to either misassembly of the structure or polarity affecting the expression of downstream genes.

## DISCUSSION

The abundance of genome sequence data has facilitated the application of reverse genetics to study the function of predicted genes in bacteria. Genetic manipulation of model organisms, such as *E. coli*, is achieved with relative ease primarily due to the “domestication” of strains by removal of restriction-modification (R/M) systems which degrade foreign incoming DNA (58). Manipulation of wild-type bacteria with medical or industrial importance is hampered both by a lack of tools and the presence of multiple R/M systems, which makes transformation and conjugation inefficient when using DNA derived from *E. coli*. In *C. difficile* 630, there are at least five type II R/M systems and one type IV restriction system (41), which partially explains the difficulties in introducing unmodified DNA into this strain.

A predominant method for generating mutations in *C. difficile* is the ClosTron system, which was originally designed to make insertional mutations, but this is limited by the number of selectable markers subsequently available to produce multiple mutants (59). Alternative methods to produce markerless deletions either require prior mutagenesis of strains, e.g., *pyrE* mutants to allow selection of integrants using 5-fluoroorotic acid (5-FOA) (60), or screening of multiple colonies to identify spontaneous resolution of integrants (61). A novel allelic exchange procedure using CRISPR-Cas9 has been described (62), but each deletion will require cloning of regions of homology and an appropriate single guide RNA (sgRNA). The frequency of spontaneous resolution for regions of homology between 300 and 600 bp have been reported to be low and inconsistent (61), presumably reflecting the stochastic nature of DNA damage or replication errors that occur within the repeat regions, which subsequently require recombination for repair. In our experiments, we were unable to detect resolution of merodiploids that contained 500-bp regions of homology unless I-SceI was expressed in the cells. Using this method with induction of double-strand breaks, we were able to generate a large deletion covering the putative *addAB* genes and a deletion of the *fliC* gene. The heterodimer of AddAB is involved in processing of double-strand breaks in a number of Gram-positive and Gram-negative bacteria. Our previous single-molecule observations for AddAB from *B. fragilis* demonstrated a translocation rate of 250 bp per s with up to 40 kb being unwound and degraded from a double-strand end (53). The sensitivity of our Δ*addAB* mutant strains to UV light and metronidazole is consistent with the encoded proteins performing the same functions in *C. difficile*. The model organism *E. coli* is the paradigm for homologous recombination and the SOS response in prokaryotes. Regulation of the SOS response in other bacteria has been relatively understudied in comparison. In *C. difficile*, the SOS regulon is controlled by a LexA homologue which modulates not only responses to DNA damage, but also regulates other processes, including motility and biofilm formation (54). Induction of the SOS response in *E. coli* requires the generation of single-stranded DNA which activates RecA to facilitate self-cleavage by LexA (63, 64). This single-stranded DNA is produced by the action of the RecBCD complex preferentially degrading one DNA strand; therefore, *recBCD* mutants do not show an SOS response (65). In *B. subtilis*, the SOS response is reported to be greatly reduced in the absence of AddAB (66). In contrast, our *C. difficile* Δ*addAB* mutant strains demonstrated activation of SOS, as indicated by cell filamentation when grown in subinhibitory concentrations of metronidazole. These data suggest there is potential redundancy in the pathway and that another exonuclease is responsible for generating single-stranded DNA from a double-strand break in the absence of AddAB. One potential candidate could be the single-stranded exonuclease RecJ, since *recJ* mutations increase the sensitivity of *addAB* mutants to DNA-damaging agents in *B. subtilis* (67). The action of other proteins on double-strand breaks in the absence of AddAB would also be consistent with the minor effect of the *addAB* deletion on the growth rate of our strains. This is also in contrast to *addAB* mutants of *B. subtilis*, which show a 50% reduction in viability (68).

The second target for demonstrating the utility of I-SceI-induced double-strand breaks was *fliC*. The deletion of *fliC* led to the expected phenotype of loss of motility, as reported previously for insertional mutations generated using the ClosTron system (57). Complementation of the Δ*fliC* mutation enabled the production of flagella, as visualized by TEM; however, the strains were not able to penetrate soft agar, which indicated a defect in flagellar function. One possible explanation is that overexpression of FliC had a detrimental effect on flagellar assembly or function in the Δ*fliC* mutant strain, although overexpression in the parental strain had no apparent effect. The *fliC* deletion in our strains extended 21 bp beyond the stop codon of the reading frame. The next downstream gene (CD630DERM_RS01755) is a glycosyltransferase which is separated from *fliC* by 92 bp. Flagella in *C. difficile* are posttranslationally modified with *N*-acetylhexosamine by the action of the glycosyltransferase encoded downstream of *fliC*. Insertional disruption of the gene RS01755 using ClosTron produced cells with reduced numbers of flagella and a nonmotile phenotype (69). The canonical −10 (TATAGT) promoter sequence of gene RS01755 is identifiable, while the putative −35 sequence (TTATTC) is more divergent from the consensus. The deletion spanning *fliC*, however, does not disrupt the putative RNA polymerase binding site for RS01755. We therefore suggest that our Δ*fliC* mutant strain is also defective in modification of flagellin as a consequence of reduced or altered expression of the glycosyltransferase. While one advantage of markerless deletions is the reduced possibility of polarity, in this case, deletion of 21 bp after the *fliC* gene exposed a potential regulatory sequence which is involved in controlling or modulating expression of the downstream gene.

Together, these results demonstrate that induction of double-strand breaks by expression of I-SceI is a useful and generally applicable tool for generating markerless deletions in *C. difficile*. The absence of the 18 bp I-SceI recognition sequence in all *Clostridium* species, and the ability of plasmids with pBP1 and pCB102 origins of replication to function in diverse clostridia, makes this approach suitable for a wide range of important organisms. Additional advantages are that amplification and cloning of large regions of homology, and counterselection, are not required, which will make the generation of deletions in wild-type strains more practical.

## MATERIALS AND METHODS

### Bacterial strains and growth conditions.

*C. difficile* 630Δ*erm* (70) was used for mutagenesis throughout this study. Cultures were routinely grown at 37°C in an atmosphere containing 10% CO_2_, 10% H_2_, and 80% N_2_ within an anaerobic cabinet (Don Whitley). The medium used for cultivation of *C. difficile* was either brain heart infusion (BHI) or anaerobic investigation medium (AIM), while LB was used for *E. coli*. Antibiotics were used at the following concentrations: 10 μg/ml erythromycin, 15 μg/ml thiamphenicol, 250 μg/ml d-cycloserine, and 8 μg/ml cefoxitin for *C. difficile*, and 500 μg/ml erythromycin and 30 μg/ml chloramphenicol for *E. coli*. The plasmids used in this study are shown in Table 1.

**TABLE 1:** Plasmids used in this work

Plasmid	Description^*a*^	Reference or source
pJIR1456	*E. coli-C. perfringens* shuttle vector (pIP404 replicon, *catP* marker), Tm^r^ Cm^r^	49
pMTL83151	*E. coli-Clostridium* shuttle vector (pCB102 replicon, *catP* marker), Tm^r^ Cm^r^	55
pMTL82254	*E. coli-Clostridium* shuttle vector (pBP1 replicon, *ermB* marker), Erm^r^	55
pMTL84151	*E. coli-Clostridium* shuttle vector (pCD6 replicon, *catP* marker), Tm^r^ Cm^r^	55
pES185	pJIR1456 + I-SceI recognition site (SacI site), Tm^r^ Cm^r^	This study
pES271	pES185 + *addBA* deletion cassette (SphI site), Tm^r^ Cm^r^	This study
pES242	pMTL83151 + I-SceI recognition site (SacI site), Tm^r^ Cm^r^	This study
pES2921	pES242 + *fliC* deletion cassette (FspI site), Tm^r^ Cm^r^	This study
pES288	pMTL82254 + P*fdx*::*I-sceI* (SbfI site), Erm^r^	This study
pES196	pMTL84151 + *fliC* gene and native promoter (NotI/XhoI site), Tm^r^ Cm^r^	This study

a Tm^r^, thiamphenicol resistant; Cm^r^, chloramphenicol resistant.

### Oligonucleotides.

A list of oligonucleotides used to generate deletions is given in Table 2. PCR amplicons representing deletion products were ligated into the appropriate vectors following digestion with the relevant restriction endonuclease using standard procedures.

**TABLE 2:** Sequences of oligonucleotides used to generate amplicons for allelic replacement vectors pES271 and pES2921

Primer	Sequence
SphI_addBA1	TTCCGCATGCTAAATGGGGATATAATACAGGC
addBA2	CCTAAGTCCCATAAATTTCCG
addBA2_addBA3	CGGAAATTTATGGGACTTAGGTGGAGTTGATGAAGCTGTTTG
SphI_addBA4	TTCCGCATGCTAGCAACCACAATATTTTCTCC
SphI-fliC1	TTCCGCATGCTTCAGCTTTAGAGTCTTTGTTG
fliC2	CTCCTTAGTATAGTTGACATCC
fliC3	GGATGTCAACTATACTAAGGAGAAAAGAAAGGATAAGGCTTTGC
SphI-fliC4	TTCCGCATGCTGGTTGTTCATGAACTTTCCC
cmFliCFor^*a*^	CCCTGGCGGCCGCAACTTTATGATAGTATGGAGC
cmFliCRev^*a*^	CCCTGCTCGAGCTATCCTAATAATTGTAAAACTC

a Primers cmFliCFor and cmFliCRev were used to produce the *fliC* complementing plasmid pES196.

### Conjugation of plasmids into *C. difficile*.

Deletion constructs and the plasmid expressing I-SceI were introduced into *C. difficile* by conjugation from *E. coli* S17-1λ*pir* using a modification of a previously described method (59). Briefly, exponential cultures of donor and recipient cells were centrifuged, washed, and resuspended in a 1/10 volume of fresh medium. Different donor-to-recipient ratios were mixed, spread onto BHI agar plates, and incubated anaerobically for 16 to 24 h. The conjugation mixture was harvested in prereduced phosphate-buffered saline (PBS) and spread onto plates containing thiamphenicol, d-cycloserine, and cefoxitin.

### Metronidazole and UV sensitivity tests.

Cultures of control and deletion strains were grown to an optical density at 600 nm (OD_600_) of 0.3 in AIM and serially diluted in prereduced PBS. Five-microliter aliquots were then spotted onto AIM plates. For metronidazole sensitivity, plates contained 0.25, 0.125, or 0.06 μg/ml metronidazole. For UV sensitivity, plates were irradiated at 10, 15, or 20 J/m^2^. All plates were incubated anaerobically for 48 h, and all experiments were performed in triplicate.

### Motility assay.

An inoculum from a single colony of each strain was stabbed into a glass tube containing AIM with 0.175% agar. The swim agar tube was incubated anaerobically at 37°C for 24 h.

### Microscopy and transmission electron microscopy.

For phase-contrast microscopy, cells were fixed with 20% formaldehyde before being placed on slides coated with poly-l-lysine. Images were captured on a Metalux II microscope using a Hamamatsu digital camera with the Improvision Openlab software. For TEM, a colony of each strain was resuspended in prereduced PBS before the addition of 3% glutaraldehyde. A 20-μl sample was placed on a carbon-coated grid followed by staining with 1% phosphotungstic acid. Grids were examined using a Philips CM120 BioTWIN transmission electron microscope.
